# Improved DC and RF Characteristics of GaN HEMT Using a Back-Barrier and Locally Doped Barrier Layer

**DOI:** 10.3390/mi16070779

**Published:** 2025-06-30

**Authors:** Shuxiang Sun, Lulu Liu, Gangchuan Qu, Xintong Xie, J. Ajayan

**Affiliations:** 1 Zhumadian Key Laboratory of Novel Semiconductor Devices and Reliability, Huanghuai University, Zhumadian 463000, Chinagalant10@163.com (G.Q.); 2 School of Electronic Science and Engineering, University of Electronic Science and Technology of China, Chengdu 610054, China; 3 Department of Electronics and Communication Engineering, SR University, Warangal 506371, India

**Keywords:** GaN HEMT, DC and RF characteristics, back-barrier layer, locally doped barrier layer

## Abstract

To enhance the DC and RF performance of AlGaN/GaN HEMTs, a novel device structure was proposed and investigated through simulation. The key innovation of this new structure lies in the incorporation of an Al_0.7_In_0.15_Ga_0.15_N back-barrier layer and an N-type locally doped AlGaN barrier layer (BD-HEMT), based on conventional device architecture. The Al_0.7_In_0.15_Ga_0.15_N back-barrier layer effectively confines electrons within the channel, thereby increasing the electron concentration. Simultaneously, the N-type locally doped AlGaN barrier layer introduced beneath the gate supplies additional electrons to the channel, further enhancing the electron density. These modifications collectively lead to improved DC and RF characteristics of the device. Compared to the conventional AlGaN/GaN HEMT, BD-HEMT achieves a 24.8% increase in saturation drain current and a 10.4% improvement in maximum transconductance. Furthermore, the maximum cutoff frequency and maximum oscillation frequency are enhanced by 14.8% and 21.2%, respectively.

## 1. Introduction

As a third-generation semiconductor material, GaN offers several significant advantages, including a wide bandgap, high electron saturation velocity, high breakdown electric field, and excellent thermal conductivity [1,2,3]. Owing to these properties, GaN-based high-electron-mobility transistors (HEMTs) exhibit outstanding performance in high-frequency, high-voltage, and high-temperature applications, making them a key component in modern electronic systems [4,5,6].

For AlGaN/GaN HEMTs, the concentration of two-dimensional electron gas (2DEG) at the heterojunction is a key factor influencing the device’s DC and frequency characteristics [7]. Therefore, appropriately increasing the 2DEG concentration can effectively enhance both the DC and RF performance of AlGaN/GaN HEMTs. To achieve this, several strategies can be employed, such as increasing the aluminum (Al) content in the AlGaN layer, optimizing the AlGaN layer thickness, and inserting an AlN interlayer at the AlGaN/GaN interface [8,9,10]. However, an excessively high Al composition may exacerbate lattice mismatch, increase defect density, and compromise device reliability. Similarly, an overly of a thin AlGaN layer can result in a higher gate leakage current and reduced breakdown voltage. In addition, while the AlN interlayer can improve confinement, it may also introduce interface scattering, which negatively affects the high-frequency performance. Non-uniform thickness in the epitaxially grown AlN layer may further lead to reduced electron mobility. Hence, it is essential to explore alternative approaches that can effectively increase 2DEG concentration while maintaining a balanced trade-off among other critical device characteristics.

For AlGaN/GaN HEMTs, employing a quaternary alloy material layer as a back-barrier can effectively confine electrons within the GaN channel, thereby increasing the 2DEG concentration [11], while also reducing electron scattering caused by defects in the buffer layer. Furthermore, introducing localized N-type doping in the AlGaN barrier layer beneath the gate can further enhance the 2DEG concentration [12]. Therefore, this study investigates—through simulation—the impact of combining an AlInGaN back-barrier layer with an N-type locally doped barrier layer beneath the gate on the DC and RF characteristics of AlGaN/GaN HEMTs.

## 2. Structure and Simulation Models

**Figure 1:**
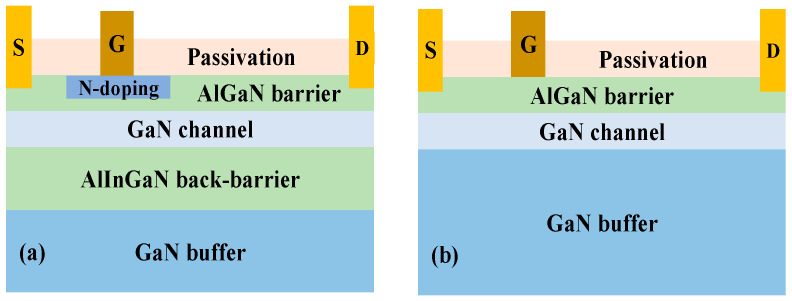
Schematic diagrams of (**a**) BD-HEMT and (**b**) C-HEMT.

Figure 1a and 1b show the schematic structures of the GaN HEMT with an Al_0.7_In_0.15_Ga_0.15_N back-barrier layer and an N-type locally doped barrier layer (BD-HEMT) and the conventional GaN HEMT (C-HEMT), respectively. In BD-HEMT, the N-type locally doped barrier layer has a doping concentration of 1 × 10^18^ cm^−3^ [13], a thickness of 20 nm, and a length of 2.1 μm. The source-to-gate spacing is set to 1.5 μm, the gate-to-drain spacing to 2.4 μm, and the gate length to 1.1 μm. Both the source and drain regions are heavily doped with an N-type concentration of 1 × 10^20^ cm^−3^ to ensure good ohmic contact. The GaN buffer layer is background-doped at 1 × 10^16^ cm^−3^, and the gate work function is set to 5.2 eV [14]. All other structural parameters of BD-HEMT are identical to those of the C-HEMT, as listed in Table 1.

**Figure 2:**
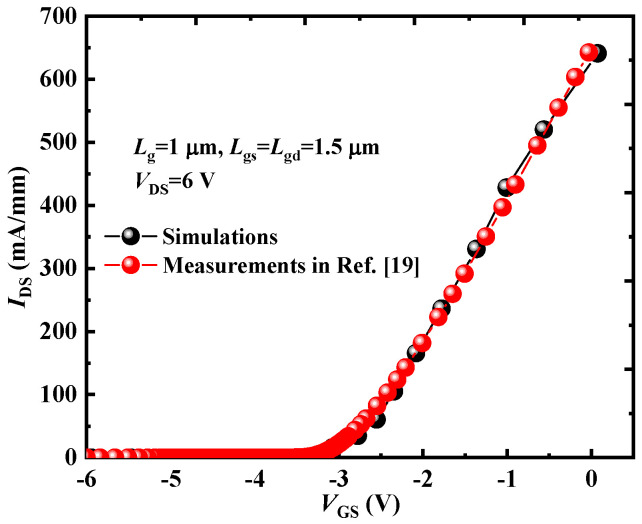
Comparison of the simulated and experimental transfer characteristics.

The simulation results of the device characteristics were obtained using the Sentaurus-TCAD. In the simulations, several physical models were employed, including the hydrodynamic model, the doping-dependent and high-field-dependent mobility model, and the Shockley–Read–Hall (SRH) recombination model [15,16,17,18]. To validate the accuracy of these models, the simulated transfer characteristics were compared with experimental measurement data [19], as shown in Figure 2. The results demonstrate excellent agreement between the simulated and measured transfer and output characteristics.

## 3. Results and Discussion

The mole fraction of Al_0.7_In_0.15_Ga_0.15_N back-barrier is taken from reference [20]. In the simulation, the drain voltage (*V*_DS_) was fixed at 8 V, while the gate voltage (*V*_GS_) was swept from −6 V to 1 V.

**Figure 3:**
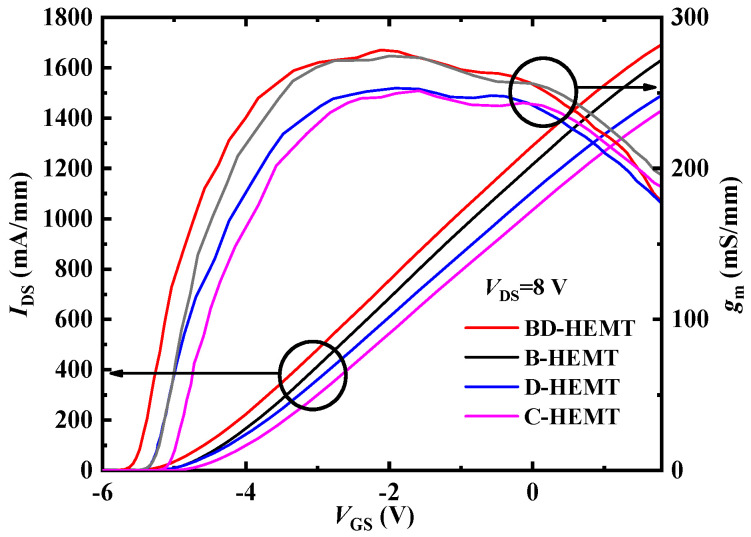
Transfer characteristics of BD–HEMT, B–HEMT, D–HEMT, and C–HEMT.

**Figure 4:**
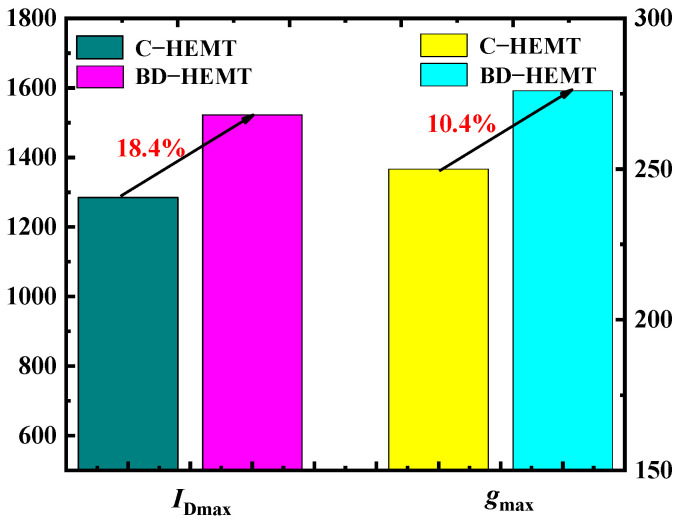
*I*_Dmax_ and *g*_max_ of BD–HEMT and C–HEMT.

Figure 3 presents the transfer characteristics of BD-HEMT, B-HEMT (with only the AlInGaN back-barrier layer), D-HEMT (with only the locally doped AlGaN barrier layer), and C-HEMT. As evident from Figure 3, the highest drain current (*I*_DS_) and transconductance (*g*_m_) are achieved for BD-HEMT. Figure 4 displays the extracted maximum drain current (*I*_Dmax_) and maximum transconductance (*g*_max_) values from Figure 3. At *V*_GS_ = 1 V, the BD-HEMT achieves an *I*_Dmax_ of 1522 mA/mm, representing an 18.4% improvement compared to the C-HEMT’s 1285 mA/mm. Furthermore, BD-HEMT exhibits a *g*_max_ of 278 mS/mm, which is 10.4% higher than the C-HEMT’s 251 mS/mm. DIBL is a key parameter for evaluating the electrostatic integrity of HEMTs. It quantifies the threshold voltage shift induced by variations in the drain voltage. A lower DIBL value indicates superior gate control and enhanced immunity to short-channel effects [21]. The DIBL of 84 mV/V for BD-HEMT is obtained using 87 mV/V for C-HEMT. Therefore, BD-HEMT shows a better gate control ability.

**Figure 5:**
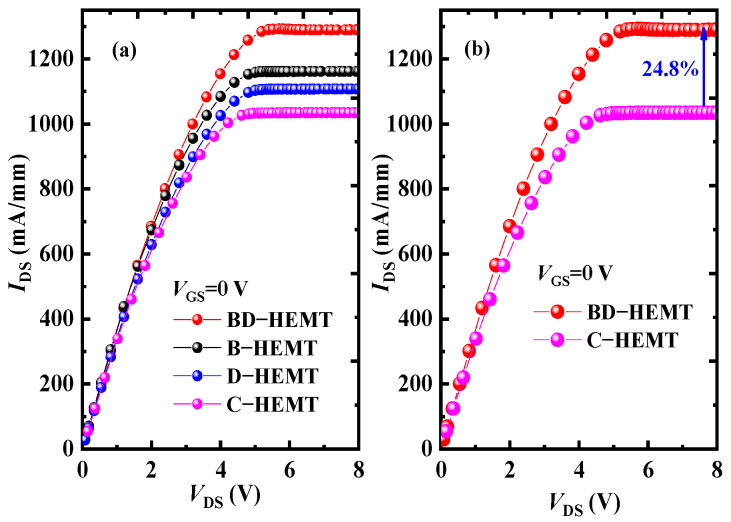
(**a**) Output characteristics for different devices. (**b**) Output characteristics for BD–HEMT and C–HEMT.

Figure 5a shows the output characteristics of BD–HEMT, B–HEMT, D–HEMT, C–HEMT, and C–HEMT, with *V*_DS_ ranging from 0 V to 8 V at *V*_GS_ values of 0 V. The results demonstrate that the incorporation of both the AlInGaN back-barrier layer and locally doped AlGaN barrier layer leads to a significant increase in *I*_DS_. At *V*_GS_ = 0 V, BD–HEMT achieves a saturation drain current (*I*_Dsat_) of 1290 mA/mm, representing a 24.8% enhancement compared to the C–HEMT’s 1035 mA/mm, as shown in Figure 5b.

**Figure 6:**
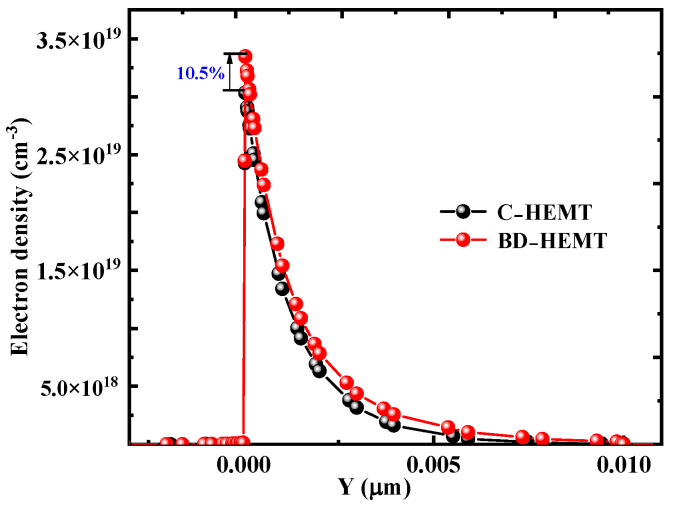
Electron distribution of BD–HEMT and C–HEMT.

**Figure 7:**
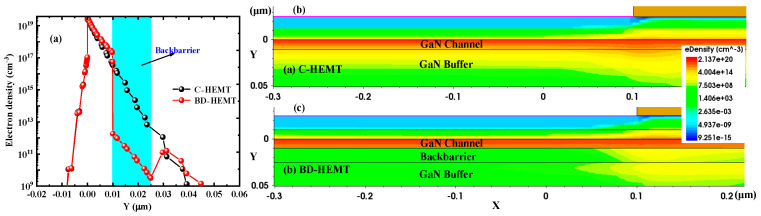
(**a**) Electron distribution of BD–HEMT and C–HEMT; two–dimensional distribution of electron concentration for (**b**) C–HEMT and (**c**) BD–HEMT.

To investigate the mechanism behind the improved *I*_Dsat_ and *g*_max_ in BD-HEMT, the electron concentration distribution in the channel was analyzed. Figure 6 presents the electron concentration profiles of BD-HEMT and C-HEMT. The results clearly show a significant increase in electron concentration within the BD-HEMT channel, with a 10.5% higher peak electron concentration compared to C-HEMT. This enhancement is attributed to the effective electron confinement in the GaN channel by the AlInGaN back-barrier layer, as demonstrated in Figure 7a. As clearly shown in the figure, the AlInGaN back-barrier layer induces a rapid decline in electron density beneath the BD-HEMT channel. In stark contrast, the C–HEMT exhibits significant electron spillage into the GaN buffer layer due to the absence of such confinement mechanism. Figure 7b,c present the two-dimensional distribution of electron concentration in BD–HEMT and C–HEMT devices. Although electrons in C–HEMT are primarily distributed in the channel, noticeable electron leakage into the GaN buffer layer is observed, as shown in Figure 7b. In contrast, electrons are effectively confined within the channel region without forming any parasitic conduction path in the GaN buffer layer for BD–HEMT, as shown in Figure 7c. These findings confirm that the incorporation of the AlInGaN back-barrier layer significantly enhances the electron concentration in the GaN channel, thereby improving the device’s DC performance characteristics.

**Figure 8:**
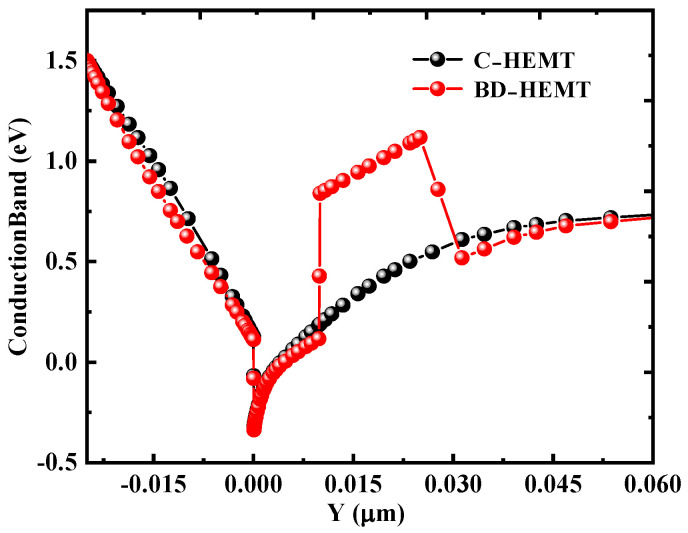
Conduction band diagrams of BD-HEMT and C-HEMT.

The AlInGaN back–barrier effectively confines electrons within the channel by introducing a conduction band offset at the GaN/AlInGaN interface. This offset forms an additional energy barrier that significantly suppresses electron leakage into the buffer layer. Figure 8 presents the conduction band diagrams of both BD–HEMT and C–HEMT. The results clearly reveal a newly formed energy band discontinuity of 0.57 eV at the interface between the GaN channel layer and the AlInGaN back-barrier. To leak from the GaN channel into the GaN buffer layer, electrons must overcome this substantially increased energy barrier [11]. Thus, the AlInGaN back–barrier layer effectively suppresses electron overflow from the channel, thereby enhancing the electron concentration within the channel.

**Figure 9:**
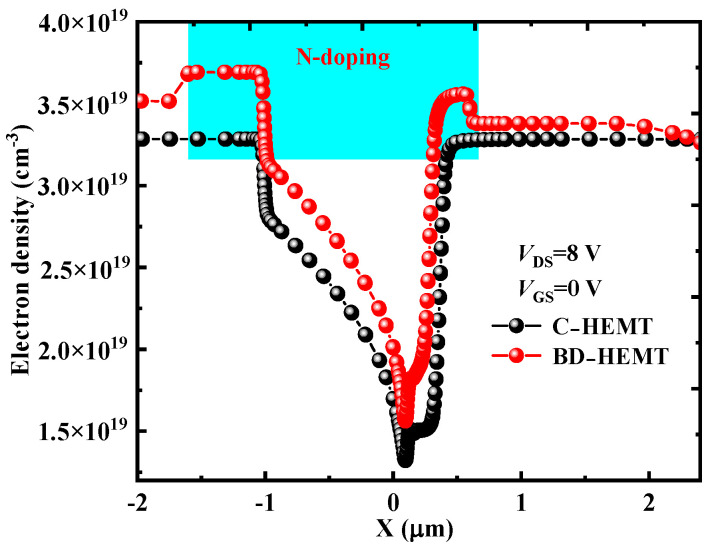
Electron concentration distribution along the GaN channel for BD-HEMT and C-HEMT.

Additionally, the N-type locally doped barrier layer supplies extra electrons to the channel, thereby further increasing the electron concentration in the channel region. Figure 9 presents the electron concentration profiles along the AlGaN/GaN interface for both BD-HEMT and C-HEMT. As clearly illustrated, the electron concentration in BD-HEMT is significantly higher than that in C-HEMT across the entire measured range, with a particularly pronounced increase observed directly beneath the N-type locally doped barrier layer. This clearly demonstrates that the N-type locally doped barrier layer effectively injects additional electrons into the GaN channel, thereby further enhancing both the *I*_Dsat_ and *g*_max_ in BD-HEMT devices.

**Figure 10:**
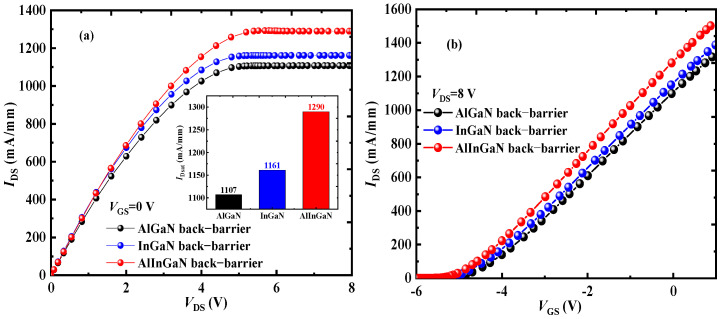
(**a**) Output and (**b**) transfer characteristics for structure with the different back-barrier materials.

Figure 10 shows the effect of the same thickness of Al_0.05_Ga_0.95_N, In_0.1_Ga_0.9_N and AlInGaN back barrier on the output characteristics of the GaN HEMT. The results show that the device with the AlInGaN back-barrier has achieved better *I*_DS_.

**Figure 11:**
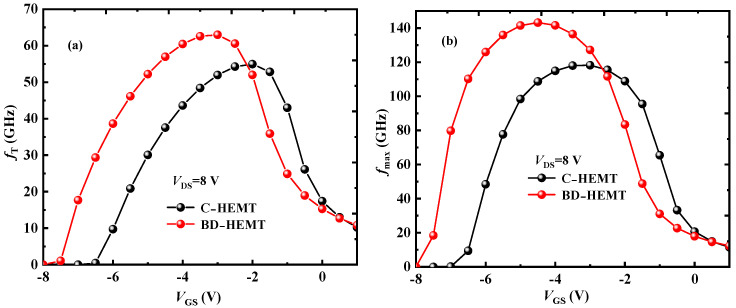
(**a**) *f*_T_ and (**b**) *f*_max_ of BD-HEMT and C-HEMT.

Figure 11 presents the variations of cutoff frequency (*f*_T_) and maximum oscillation frequency (*f*_max_) with *V*_GS_. As clearly demonstrated in the figures, BD-HEMT exhibits significant improvements in *f*_T_ and *f*_max_. The maximum *f*_T_ of BD-HEMT and C-HEMT is 63 GHz and 55 GHz, respectively, and the maximum *f*_max_ is 143 GHz and 118 GHz, respectively. Compared with C-HEMT, the maximum *f*_T_ and *f*_max_ of BD-HEMT increased by 14.8% and 21.2%, respectively.

**Figure 12:**
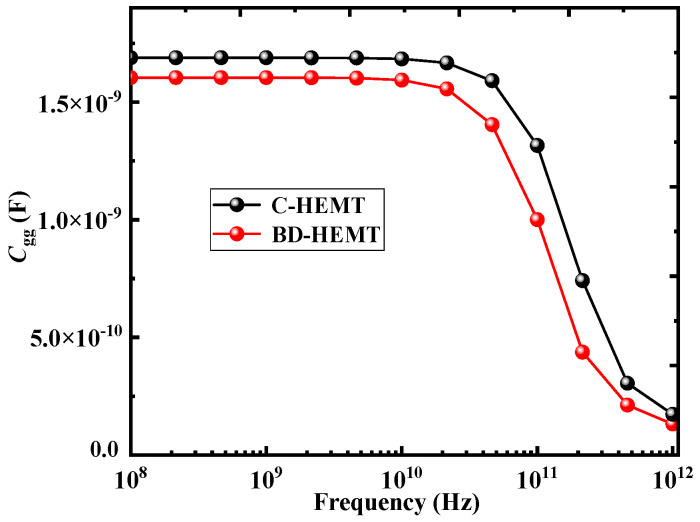
Variation of *C*_gg_ for BD-HEMT and C-HEMT.

To explain the reasons for the increase in *f*_T_ and *f*_max_ of the device after introducing the AlInGaN back-barrier layer and locally doped AlGaN barrier layer, the variation of the gate capacitance (*C*_gg_) with frequency was simulated and studied, as shown in Figure 12. The simulation results show that, compared with C-HEMT, the *C*_gg_ of BD-HEMT is smaller throughout the entire frequency range. Therefore, BD-HEMT achieves higher *f*_T_ and *f*_max_ [13]. Furthermore, the introduction of the locally doped AlGaN barrier layer increases the number of electrons beneath the gate-source and gate-drain regions, thereby resulting in a decrease in the gate-source and gate-drain resistances, which further increases *f*_T_ and *f*_max_.

## 4. Conclusions

In this paper, the DC and RF characteristics of BD-HEMT are studied through simulation. The simulation results show that the DC and RF characteristics of the device are significantly improved after the introduction of AlInGaN back-barrier layer and N-type locally doped AlGaN barrier layer. Compared with C-HEMT, BD-HEMT improves in *I*_Dsat_ by 24.8%, *g*_max_ by 10.4%, maximum *f*_T_ and *f*_max_ by 14.8% and 21.2%, respectively. This result is caused by the combination of the AlInGaN back-barrier layer and the locally doped AlGaN barrier layer to increase the electron concentration in the channel.

## Figures and Tables

**Table 1:** Structural parameters of BD-HEMT.

Layer	Value
Passivation layer	50 nm
N-type locally doped barrier layer	20 nm
Al_0.3_Ga_0.7_N barrier layer	25 nm
GaN channel layer	10 nm
Al_0.7_In_0.15_Ga_0.15_N back-barrier layer	15 nm
GaN buffer layer	1.5 μm

## Data Availability

Data are contained within the article.
